# The patient clinical journey and socioeconomic impact of osteogenesis imperfecta: a systematic scoping review

**DOI:** 10.1186/s13023-023-02627-3

**Published:** 2023-02-22

**Authors:** Maria Rapoport, Michael B. Bober, Cathleen Raggio, Lena Lande Wekre, Frank Rauch, Ingunn Westerheim, Tracy Hart, Taco van Welzenis, Arun Mistry, James Clancy, Lucy Booth, Samantha Prince, Oliver Semler

**Affiliations:** 1 Wickenstones Ltd, Abingdon, Oxfordshire UK; 2 Alfred I. duPont Hospital, Wilmington, Delaware USA; 3 Hospital for Special Surgery, New York, USA; 4 TRS National Resource Center for Rare Disorders, Sunnaas Rehabilitation Hospital, Bjørnemyr, Nesodden Norway; 5 McGill University, Montreal, Canada; 6 Osteogenesis Imperfecta Federation Europe, Heffen, Belgium; 7 Osteogenesis Imperfecta Foundation, Gaithersburg, MD USA; 8 Mereo Biopharma, London, UK; 9 University of Cologne, Cologne, Germany

**Keywords:** Osteogenesis imperfecta, Health-related quality of life, Clinical, Economic, Caregiver, Adult, Paediatric

## Abstract

**Background:**

Osteogenesis imperfecta (OI) is a rare heritable connective tissue disorder primarily characterised by skeletal deformity and fragility, and an array of secondary features. The purpose of this review was to capture and quantify the published evidence relating specifically to the clinical, humanistic, and economic impact of OI on individuals, their families, and wider society.

**Methods:**

A systematic scoping review of 11 databases (MEDLINE, MEDLINE in-progress, EMBASE, CENTRAL, PsycINFO, NHS EED, CEA Registry, PEDE, ScHARRHUd, Orphanet and Google Scholar), supplemented by hand searches of grey literature, was conducted to identify OI literature published 1st January 1995–18th December 2021. Searches were restricted to English language but without geographical limitations. The quality of included records was assessed using the AGREE II checklist and an adapted version of the JBI cross-sectional study checklist.

**Results:**

Of the identified 7,850 records, 271 records of 245 unique studies met the inclusion criteria; overall, 168 included records examined clinical aspects of OI, 67 provided humanistic data, 6 reported on the economic impact of OI, and 30 provided data on mixed outcomes. Bone conditions, anthropometric measurements, oral conditions, diagnostic techniques, use of pharmacotherapy, and physical functioning of adults and children with OI were well described. However, few records included current care practice, diagnosis and monitoring, interactions with the healthcare system, or transition of care across life stages. Limited data on wider health concerns beyond bone health, how these concerns may impact health-related quality of life, in particular that of adult men and other family members, were identified. Few records described fatigue in children or adults. Markedly few records provided data on the socioeconomic impact of OI on patients and their caregivers, and associated costs to healthcare systems, and wider society. Most included records had qualitative limitations.

**Conclusion:**

Despite the rarity of OI, the volume of recently published literature highlights the breadth of interest in the OI field from the research community. However, significant data gaps describing the experience of OI for individuals, their families, and wider society warrant further research to capture and quantify the full impact of OI.

**Supplementary Information:**

The online version contains supplementary material available at 10.1186/s13023-023-02627-3.

## Background

Osteogenesis imperfecta (OI) is a rare, heritable connective tissue disorder with multiple manifestations. Individuals with OI typically have low bone mass and skeletal fragility, and are susceptible to fractures of the long bones, vertebral compression, variable bone deformities, scoliosis and growth deficiency [1].

OI can also result in an array of secondary features including blue sclerae, hearing loss, dentinogenesis imperfecta, basilar invagination, cardiovascular and pulmonary abnormalities [1].

The condition presents as a range of phenotypes, classified according to clinical presentation, radiographic features, patterns of inheritance [2] and genetics [1]. The estimated prevalence is approximately 0.4–1.1 per 10,000 individuals based on population survey and patient registry data [3–5].

A multidisciplinary approach to the medical management of OI remains unrealised; current treatment aims are the reduction of fractures and improvement in mobility and function [6]. The only currently utilised pharmacologic interventions are bisphosphonates, which reduce bone turnover and may prevent or delay bone pain and reduce fracture rates, and analgesics, specifically for pain management. Non-pharmacological interventions comprise surgery, including rodding surgery, and physiotherapy [7, 8].

Living with OI may have a significant impact on the physical, social, and emotional wellbeing of individuals as well as their families and caregivers [9–11]. Although a sizeable body of evidence describing the impact of OI on health-related quality of life (HRQoL) exists, gaps have been identified in past records, including: an understanding of wider health concerns beyond bone health [12, 13], the impact of other manifestations of OI on HRQoL, and the impact of OI on affected family members caring for affected individuals and other family members, especially non-affected siblings. Only few studies have examined facets of the socioeconomic impact of OI on patients and their caregivers, associated costs to healthcare systems and wider society, and none currently offer a comprehensive picture of the economic impact of OI. A number of systematic literature reviews (SLRs) relating to OI have been published in the last 10 years [9, 14–23]; 1 recent review reported on the impact of OI on families [9].

To our knowledge, no scoping review has comprehensively captured the breadth of the published evidence and data gaps relating to the OI patient journey. Therefore, the aim of this systematic scoping review is to capture the breadth of literature describing the clinical, HRQoL and economic impact of OI on individuals with OI, their families and caregivers, and wider society.

## Methods

A systematic scoping review of the literature was performed following Centre for Reviews and Disseminations (CRD) systematic review guidance and is reported according to the Preferred Reporting Items for Systematic Reviews and Meta-Analyses (PRISMA) recommendations [24, 25]. The protocol of this review has been registered with PROSPERO (registration number CRD42021225786). The data synthesis focussed on the scope of the literature following JBI recommendations for scoping reviews [26].

This systematic scoping review consisted of 3 review questions: What is the patient clinical journey as experienced by people living with OI?, What is the humanistic impact of OI as experienced by people living with OI, their families, and caregivers?, and What is the economic impact of OI as experienced by people living with OI, their families, caregivers, and healthcare providers?.

### Literature searches

Eleven databases were searched for relevant records published between 1st January 1995–18th December 2021: MEDLINE, MEDLINE In-Process, EMBASE, Cochrane Central Register of Controlled Trials (CENTRAL), PsycINFO, National Health Service Economic Evaluation Database (NHS EED), Center for the Evaluation of Value and Risk in Health (CEA registry), Paediatric Economic Database Evaluation (PEDE), School of Health and Related Research Utilities Database (ScHARRHUd), Orphanet and Google Scholar. Additional manual searches of grey literature were performed. The search terms are presented in the Additional file 1: materials (Additional file 1: Table S1).

### Selection of eligible studies

English language records were included if they examined the clinical impact or patient journey of adults or children with OI, the humanistic impact of OI on adults, children, or their families and caregivers, or the economic impact on individuals with OI, families and caregivers of people with OI and wider society. Primary outcomes of interest included key clinical events and health conditions, wider health concerns beyond fractures; equity concerns; socio-economic mediators for access to treatment; diagnosis and monitoring; interactions with the healthcare system; disease specific and generic HRQoL outcomes; utility measures; factors affecting HRQoL; patient reported outcomes; direct and indirect healthcare costs; healthcare resource use (HCRU); and non-healthcare costs. To answer the clinical review questions, clinical guidelines; patient registry data; patient and healthcare provider surveys; cohort studies (≥ 50 patients); cross-sectional studies (≥ 50 patients) and case–control studies (≥ 50 patients) were included. For the humanistic and economic sections, randomised-controlled trials (RCTs); non-RCTs; cohort studies; patient registry data; patient survey data; cross-sectional studies; case–control studies; case series (≥ 10 patients); economic evaluations; and HCRU or cost studies were included. A comprehensive description of eligibility criteria is provided in the Additional file 1: materials (Additional file 1: Table S2).

Duplicates were removed using Endnote algorithms and a manual screening by 1 reviewer, who also rapidly screened all titles within Endnote to remove records that clearly did not meet the eligibility criteria. At all screening stages, duplicate records, such as interim records or congress proceedings of research for which full text records were also identified, were excluded to minimise reporting bias.

Titles and abstracts of the remaining records were screened by 2 independent reviewers to identify potentially relevant records. Disagreements were resolved independently by a third reviewer. Full texts of all remaining records were screened in the same manner.

### Data extraction

The following categories of data were extracted from the included studies: record identifiers, publication type, aim of the publication/study, study design, inclusion and exclusion criteria, recruitment procedures, participant characteristics (age, sex, ethnicity, country, socio-economic status, disease characteristics, comorbidities, diagnosis method), study setting (country and venue type) and outcome data or results (unit of assessment/analysis, characteristics of each pre-specified outcome, type of analysis, results of study analysis, any additional outcomes).

### Quality assessment

Guidelines were assessed with the AGREE II (Appraisal for Guidelines Research and Evaluation II) checklist [27]. All other records were assessed using a custom tool adapted from the JBI (Joanna Briggs Institute) checklist for cross-sectional studies [28] (Additional file 1: Table S3).

### Narrative synthesis

Included records were collated, combined, and summarised in a qualitative synthesis. Results were drawn together by category (OI patient journey/clinical impact, humanistic impact of OI, economic impact of OI) and observed effects and inconsistencies across studies were explored. Outcome data were grouped where possible to enable descriptive analysis. The narrative synthesis was undertaken by one author and reviewed by all co-authors.

For the purpose of this review, individuals younger than 18 years of age were considered children and such reports were grouped in the paediatric sections.

## Results

**Fig. 1:**
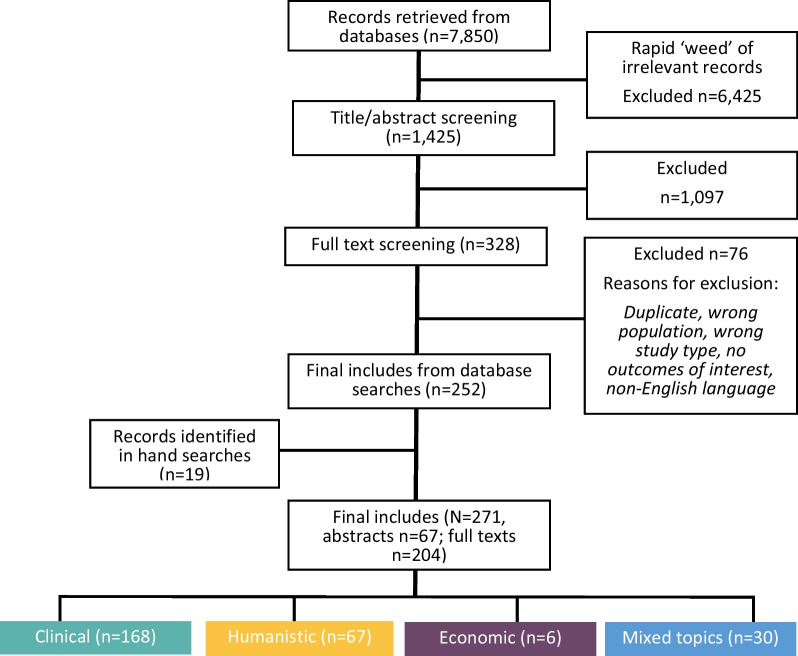
PRISMA flow diagram. *PRISMA* Preferred Reporting Items for Systematic Reviews and Meta-Analyses

The PRISMA diagram for the selection of eligible records is shown in Fig. 1. Overall, 271 records (67 abstracts, 204 full texts) of 245 unique studies met the inclusion criteria. Most records reported on clinical conditions (*n = *168, 61.8%) (Additional file 1: Table S4), or humanistic outcomes (*n = *67, 24.6%) (Additional file 1: Table S5). Only 6 records reported on economic outcomes (2.2%) (Additional file 1: Table S6). Additionally, 30 (11.1%) records reported on mixed topics (Additional file 1: Table S7).

### Clinical events and conditions in individuals with OI

**Fig. 2:**
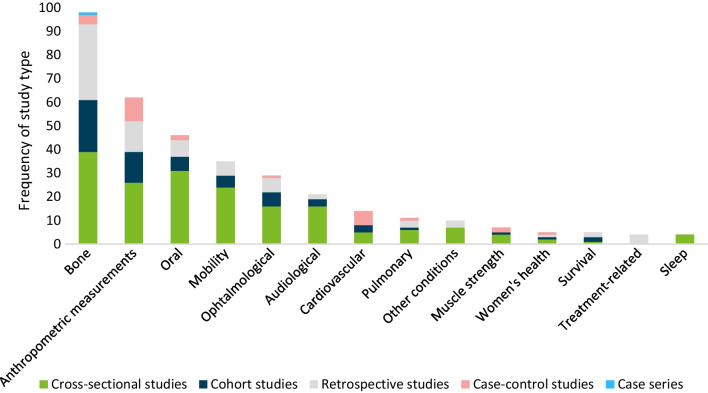
Study types by clinical condition or event in individuals with OI. *OI* Osteogenesis imperfecta

Overall, 171 records of 153 unique studies (47 abstracts, 124 full texts) on clinical events and conditions were included (Table 1). A detailed narrative synthesis of included records is presented in Table 1. Studies mostly used a cross-sectional design (Fig. 2). Data on children were included in 71 records (41.5%), 40 records (23.4%) included adults, and 55 (32.2%) included mixed populations. In 5 (2.9%) records the study population was unclear. Most well-described themes included bone, joint and musculoskeletal conditions (*n = *97, 56.7%), anthropometric measures (*n = *62, 36.3%), oral conditions (*n = *45, 26.3% of records), mobility (*n = *34, 19.9%), audiological conditions (*n = *21, 13.5%), ophthalmological conditions (*n = *29, 17.0%), cardiovascular conditions (*n = *15, 8.8%) and pulmonary conditions (*n = *11, 6.4%).

**Table 1:** Narrative synthesis of included records on clinical events and conditions

Condition	N	Findings	Records
Bone, joint, and musculoskeletal	97	Widely described in the literature, across various geographies and populations and prevalent in individuals with OI. Fractures (*n = *69), general skeletal deformity (*n = *17), scoliosis/kyphosis (*n = *28) and hypermobility (*n = *12) were well described, few records included information on basilar invagination (*n = *7), Wormian bones (*n = *10), chest deformities (*n = *4), acetabular protrusion (*n = *2), sprains (*n = *1) and dislocations (*n = *1)	[13, 30–32, 34, 37, 40, 43, 46, 78–165]
Anthropometric measures	62	Mostly reported in the paediatric population, including height (*n = *54), weight (*n = *35), and BMI (*n = *17). Fewer records of cephalometric measurements (*n = *12), relative arm span (*n = *7), body surface area measurements (*n = *6), tibia length (*n = *1) and skinfold thickness (*n = *1)	[13, 31, 37, 46, 79, 80, 83, 93, 95, 96, 98, 99, 101, 103, 112–115, 121, 122, 124, 125, 129, 131–134, 139, 142, 143, 149, 151, 154, 158, 160–163, 166–189]
Oral	45	Most described dentinogenesis imperfecta (*n = *42). Few records included caries (*n = *3), agenesis (*n = *3), taurodontism (*n = *3), temporomandibular problems (*n = *4) and occlusions (*n = *9)	[29, 31, 79, 85, 86, 89, 90, 96, 97, 100, 101, 104, 111, 115, 116, 121–124, 132–134, 153, 155, 163, 179, 190–209]
Mobility	34	Ambulation is well described (*n = *29). Fewer records provided data on motor development (*n = *14), age of sitting (*n = *2), age of gait acquisition (*n = *4) and abnormal motor development (*n = *4)	[13, 34, 43, 82, 90, 92, 95, 97, 105–108, 110–112, 116, 122, 124, 125, 129, 140, 147, 151, 163, 164, 180, 185–188, 210–213]
Ophtalmological	29	Prevalence of grey and blue sclera is well documented (*n = *27). Few records described other ophthalmological conditions (*n = *1) and vision problems (*n = *2)	[13, 31, 79, 81, 85, 86, 89, 90, 96, 99–101, 103, 111, 115, 116, 121–124, 132–134, 139, 155, 158, 163, 190, 214]
Audiological	21	Hearing loss is well-described (*n = *23) and reported to be prevalent in individuals with OI. Few records of conditions unrelated to hearing loss were identified (*n = *2). Most records included adults	[13, 29, 31, 89, 90, 93, 96, 101, 106, 111, 115, 116, 122, 123, 190, 212, 215–219]
Cardiovascular	15	Records provided clinical heart measurements (*n = *11) and information on valvular disease (*n = *8), while heart failure (*n = *2), hypertension (*n = *3) and atrial fibrillation (*n = *1) were not well-described	[13, 29, 139, 142, 151, 170, 175, 182–184, 220–224]
Pulmonary	11	Four records described breathing problems and respiratory disease. Few data on wheezing (*n = *2) and respiratory arrest (*n = *1) were identified. In most records which provided participants’ OI type it was self-reported (*n = *5) which may limit the generalisability of the identified data	[13, 46, 73, 98, 106, 142, 143, 151, 212, 225, 226]
Other conditions	10	Records described bruising and skin conditions (*n = *4), GI tract issues (*n = *2), kidney stones (*n = *3), neurological problems (*n = *2), diabetes (*n = *1), and abnormal platelet counts (*n = *1)	[13, 96, 98, 103, 106, 145, 205, 227–229]
Muscle strength	7	Individuals with OI may have lower muscle strength compared with the overall population; however, all records were conducted in children with OI. No records of muscle strength in adults were included	[82, 108, 164, 171, 210, 213, 230]
Women’s health	5	Records evaluated pregnancy and birth, suggesting that women with OI may experience higher risk pregnancies compared with other women and may be at a high risk of fracture during pregnancy and post-partum. Women with OI may be more likely to deliver by caesarean section compared with the overall population. No records of other women’s health issues were identified	[39, 45, 106, 119, 162]
Survival	5	Individuals with more severe OI types are more likely to experience excess mortality. Leading causes of death in the OI population were respiratory and cardiovascular conditions. No records of mortality, life expectancy and survival from non-western or Northern European regions were included	[29, 73, 74, 231, 232]
Treatment-related	4	All records provided data on long-term treatment-related conditions and events in children, but not in adults	[37, 118, 233, 234]
Sleep ^a^	4	Sleep conditions were reported to be prevalent in individuals with OI	[13, 106, 187, 235]

*BMI* Body mass index, *GI* Gastrointestinal, *OI* Osteogenesis imperfecta

^a^Including sleep disturbance and sleep apnoea

Less commonly described conditions included diabetes (*n = *1, 0.1%), abnormal platelet counts (*n = *1), gastrointestinal (GI) tract issues (*n = *2, 1.2%), neurological problems (*n = *2, 1.2%), kidney stones (*n = *3, 1.8%), bruising and skin conditions (*n = *4, 2.3%), treatment-related conditions (*n = *4, 2.3%), sleep-related conditions (*n = *4, 2.3%), women’s health (*n = *5, 2.9%), survival (*n = *5, 2.9%) and muscle strength (*n = *7, 4.1%).

### Diagnosis and monitoring

The diagnosis of OI was discussed in 36 records of 34 unique studies (10 abstracts, 26 full texts) (Table 2). Children were included in 15 records (41.7%), mixed populations in 16 (44.4%), adults in 3 (8.3%) [29–31] and an unclearly defined population in 1 (2.8%). One publication (2.8%) did not include a patient population. Best described were diagnostic techniques, including clinical history or radiographic assessment (*n = *20), genetic testing (*n = *10), and dual-energy X-ray absorptiometry (DEXA) scans (*n = *7). Fewer records included other diagnostic techniques, such as skin biopsy or collagen analysis (*n = *4), blood tests (*n = *3) and prenatal diagnosis (*n = *4). Equal amounts of records reported on genetic testing in 2010–2015 and 2016–2020. Additionally, age at diagnosis (*n = *11), diagnostic pathways (*n = *5), and misdiagnosis or diagnostic uncertainty (*n = *5) were explored.

**Table 2:** Records including information on diagnosis in individuals with OI

	Skin biopsy/ collagen analysis	Blood/ DNA analysis	Genetic test (general)	Clinical history and radiographs (general)	DEXA	Prenatal testing	Diagnostic pathway	Age of diagnosis	Misdiagnosis/ diagnostic uncertainty
Aarabi, Rauch [144]				x					
Arponen [158]				x					
Arponen, Mäkitie [154]				x					
Aubry-Rozier, Richard [35]		x		x	x		x		
Bellur, Jain [40]						x		x	
Binh, Maasalu [90]				x					
Brizola, Zambrano [153]				x		x		x	
Castro, Santos [89]				x				x	
Cubert, Cheng [236]				x		x		x	
Dar, Khalily [29]			x						x
Greeley, Donaruma-Kwoh [117]				x		x		x	
Hagberg, Lowing [33]			x	x	x				
Jain, Tam [174]			x						
Kok, Sakkers [165]				x					
Liu, Asan [237]								x	
Martin, Haney [102]					x				
Moreira, Gilbert [42]									x
Narayanan, Dougan [34]					x			x	
Oduah, Firth [85]				x					
Ohata, Takeyari [121]			x	x					
Patel, Nagamani [123]			x						
Pinheiro, Barrios [184]				x					
Rauch, Lalic [134]			x						
Rusinska and Michalus [86]									x
Rusinska, Jakubowskapietkiewicz [155]			x	x	x				
Sepúlveda, Terrazas [32]				x				x	
Simoes, Fernandes [228]			x						
Song, Zhao [163]								x	
Stewart, Raja [30]					x		x		
Tabanfar [101]			x	x					x
Tosi, Floor [212]	x	x							
Tosi, Floor [106]	x	x		x					
Vyskocil and Pavelka [238]	x			x	x		x		
Wekre, Frøslie [31]							x	x	
Yimgang, Brizola [46]								x	
Youngblom, Murray [239]	x		x	x			x		x

DEXA, dual-energy x-ray absorptiometry

In comparison to records including data on diagnosis, fewer records describing the monitoring of patients with OI were included (*n = *5; 2 abstracts, 3 full texts). Such records mostly described monitoring techniques and procedures (*n = *5), including DEXA scans, vision exams, blood pressure readings, blood tests, body mass index (BMI), height or weight measurements, dental exams, bone turnover marker measurements, range of motion or patient reported measurements [13, 32–35]. One record also provided insights on monitoring frequency [35].

### Current care practice

Data on current care practice were included in 74 records of 70 unique studies (12 abstracts, 62 full texts) (Table 3). Most records reported on children (*n = *39, 52.7%); fewer reported on adults (*n = *13, 17.6%) and mixed populations (*n = *22, 29.7%). Themes included pharmacological interventions (*n = *58), surgical interventions (*n = *29), other interventions (*n = *7) and pregnancy and birth (*n = *5).

**Table 3:** Current care practice for individuals with OI

	Bisphosphonates	Vitamins	Analgesics	Blood pressure medication	Other medication	Rodding	Spine	Plate fixation	Osteosynthesis	Osteotomies	Dental	Stapes/ear surgery	Pinning	Tension band	General	Physiotherapy	Other	MoD for women with OI	MoD for foetus with OI
Tosi, Oetgen [13]		x	x	x		x													
Tosi, Floor [106]						x	x												
Najirad, Ma [203]	x																		
Najirad, Madathil [204]	x																		
Song, Zhao [163]	x																		
McKiernan [103]	x		x																
Li, Xia [99]	x	x																	
Montpetit, Lafrance [188]	x					x	x												
Atta, Iqbal [107]	x																		
Ruck, Dahan-Oliel [240]	x					x													
Engelbert, Beemer [108]						x													
Bains, Carter [130]	x																		
Hoyer-Kuhn, Semler [37]	x															x			
de Graaff, Verra [38]	x	x																	
Feehan, Zacharin [109]	x		x	x	x										x				
Vanz, van de Sande Lee [110]	x															x			
Brizola, Staub [210]	x					x													
Daly, Wisbeach [211]						x													
DeVile, Allgrove [156]	x					x	x												
Germain-Lee, Brennen [168]	x																		
Oliveira, Peters [100]	x																		
Patel, Nagamani [123]	x																		
Scheres, van Dijk [124]	x	x													x				
Trejo, Fassier [118]	x																		
Wekre, Eriksen [125]	x	x			x										x				
Apolinário, Sindeaux [195]	x																		
Arponen, Mäkitie [154]	x																		
Jain, Tam [174]	x					x													
Palomo, Glorieux [176]	x																		
Goudriaan, Harsevoort [78]						x		x											
Rauch, Plotkin [131]	x																		
Rodriguez Celin, Kruger [105]						x													
Sepúlveda, Terrazas [32]	x																		
Arponen [158]	x																		
Dar, Khalily [29]	x																		
Kadhim, Holmes [83]	x																		
Michalus, Nowicka [233]	x																		
Oduah [84]	x					x													
Oduah, Firth [85]	x					x											x		
Tosi, Floor [212]						x	x												
Yakhyaeva and Namazova-Baranova [88]	x																		
Castro, Santos [89]	x																		
Anissipour, Hammerberg [138]	x																		
Semler, Hoyer-Kuhn [186]	x															x			
Kruger, Caudill [180]	x																		
Binh, Maasalu [90]	x								x	x									
Narayanan, Dougan [34]	x	x													x				
Okawa, Kubota [200]	x										x								
Cubert, Cheng [236]																			x
Bellur, Jain [40]																			x
Payet and Cormier [91]	x	x																	
Swinnen, De Leenheer [93]	x											x							
Peddada, Sullivan [94]	x																		
Rauch, Robinson [181]	x																		
Tayne and Smith [95]	x					x		x					x	x	x		x		
Hald, Folkestad [96]												x							
Belyea and Knox [53]							x												
Engelbert, Uiterwaal [97]						x													
Tam, Chen [147]	x																		
Yimgang, Brizola [46]																			x
Yimgang and Shapiro [45]																		x	
Zambrano, Brizola [185]	x																		
Pedersen [218]												x							
McAllion and Paterson [162]																		x	
Zambrano, Brizola [129]	x	x																	
Aubry-Rozier, Richard [35]																x			
Goeller, Esposito [234]	x	x													x				
Munns, Rauch [112]	x					x										x			
Cheung, Arponen [133]	x																		
Lindahl, Kindmark [113]	x																		
Ben Amor, Roughley [132]	x																		
Sato, Ouellet [161]	x						x												
Salter, Offiah [229]	x																		
Semler, Cheung [160]	x																		
Hald, Folkestad [114]	x	x																	

*MoD* Mode of delivery

Included records on pharmacological interventions focussed on bisphosphonate use (*n = *57), while the use of other medications, including vitamins or supplements (*n = *10), analgesics (*n = *3) or blood pressure and other medications (*n = *2 each) was less well documented. Records of surgical interventions mostly included information on rodding procedures (*n = *48). Other types of surgery (*n* ≤ 7 each for all other surgery types), use of physiotherapy (*n = *5) and other non-pharmacological interventions and delivery methods (*n = *5) were not well described.

### Interactions with healthcare professionals

Of 15 unique records (4 abstracts, 11 full texts) [13, 30, 33–45], most included children (*n = *7, 46.7%); fewer records including adults or mixed populations were identified (*n = *4, 26.7% each). Records described the utilisation of services (*n = *10) [13, 30, 36–41, 45, 46], experience with services (*n = *3) [35, 41, 42], progression through the healthcare system (*n = *2) [33, 35] and interactions with specific healthcare professionals or consultants (*n = *9) [13, 33–37, 41–43]. While many records described ante- and postnatal care (*n = *4) (39) [40, 45, 46], occupational and physical therapy (*n = *4) [37, 41–43] and multidisciplinary care approaches (*n = *5) [13, 33–35], fewer records described dental care [36] and outpatient care (*n = *1 each) [38].

### Guidance for clinical practice for individuals with OI

Across 13 unique, full text records of guides for clinical practice most (*n = *8, 61.5%) were not specific to OI (Table 4). Of the 13 included records, 7 were published prior to 2013 and all guidance for clinical practice were published for Northern and Western Europe, the USA and Australia.

**Table 4:** Included records on guidance for care practice for individuals with OI

	Country	Organisation/ author	Population	Type	OI specific	Subject
Antoniazzi, Mottes [47]	Italy	Expert HCPs	Mixed	Guidelines	Yes	Practical guidelines for the treatment of OI in Italy
Bianchi, Leonard [241]	International	Taskforce of the Paediatric Position Development conference	Paediatric	Position statement	No	Review of the use of DEXA in children with chronic diseases
Byers, Krakow [242]	USA	Expert HCPs	Mixed	Guidelines	Yes	Guidelines on laboratory testing, pre-natal testing, family history and clinical detection of OI at different ages
Cianferotti and Brandi [243]	Italy	Italian Society for Osteoporosis, Mineral Metabolism and Bone Diseases	Mixed	Guidelines^a^	No	Superseded by guidelines published in 2016
Galindo-Zavala, Bou-Torrent [244]	Spain	Expert HCPs	Paediatric	Consensus guidelines	No	Expert panel consensus recommendations for the prevention, diagnosis and treatment of secondary childhood osteoporosis
Mueller, Engelbert [245]	International	Expert panel of 13^th^ Conference on Osteogenesis Imperfecta	Paediatric	Consensus statement	Yes	16 consensus statements on physical training for rehabilitation and improved motor function in children with OI
Rossini, Adami [246]	Italy	Italian Society for Osteoporosis, Mineral Metabolism and Bone Diseases	Mixed	Guidelines	No	General guidelines for management and diagnosis for both primary and secondary osteoporosis and associated diseases
Ruggiero, Dodson [247]	USA	American Association of Oral and Maxillofacial Surgeons	Mixed	Position statement	No	Strategies for the diagnosis, treatment, and management of bisphosphonate-related osteonecrosis of the jaw
Shapiro and Germain-Lee [248]	USA	Expert HCPs	Mixed	Guidelines	Yes	Effective transition from paediatric to adult OI care
Simm, Biggin [249]	Australia	APEG working group	Paediatric	Consensus guidelines	No	Consensus guidelines on the recommended use of bisphosphonates in children
van Brussel, van der Net [250]	Netherlands	Expert HCPs	Mixed	Guidelines	No	Physical activity in children with OI
van Dijk, Byers [251]	International	EMQN	Mixed	Consensus guidelines	Yes	Best practice consensus guidelines for the molecular and genetic diagnosis of OI
White, White [252]	International	Delphi panel of expert HCPs	Mixed	Consensus guidelines	No	Multidisciplinary guidelines for diagnosis, evaluation, and treatment of patients with skeletal dysplasia with spinal pathology Guidelines for management and diagnosis for both primary and secondary osteoporosis

^a^Superseded

*APEG* Australasian Paediatric Endocrine Group, *DEXA* Dual-energy X-ray absorptiometry, *EMQN* European Molecular Genetics Quality Network, *HCP* Health care professional, *OI* Osteogenesis

Most such records covered mixed populations (*n = *9, 69.2%). 4 were specific to children (30.8%). OI-unspecific records covered the diagnosis and management of osteoporosis, skeletal dysplasias or spinal pathology, exercise recommendations for children with chronic conditions, use of bisphosphonates in children, treatment of bisphosphonate-related osteonecrosis of the jaw and the use of DEXA scans in children with chronic disease.

The 5 OI-specific clinical practice guides (38.5%) covered the physical training and rehabilitation of children with OI, the transition of young adults from paediatric to adult care, and best practice for the molecular and genetic diagnosis of OI. Only 1 record published in 2000 described best management practices specific to OI [47].

### HRQoL of adults with OI

**Fig. 3:**
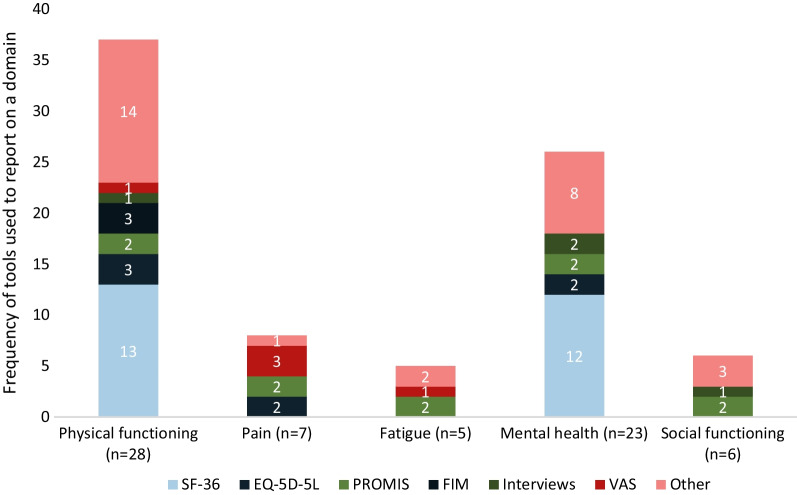
Tools used to assess adult HRQoL domains and number of records on each domain. *EQ-5D-5L* Euroqol 5-Dimension Questionnaire 5 Levels, *FIM* Functional Independence Measure, *HRQoL* Health-related quality of life, *PROMIS* Patient-reported Outcomes Measurement, *VAS* Visual Analogue Scale

Of 32 records (2 abstracts, 30 full texts) of 48 unique studies most (*n = *28) described adults (87.5%) and 4 (12.5%) mixed populations. Across studies that provided the sex of participants (*n = *31), women were overrepresented (median: 59.0%). Few records focussed on young or older adults. No OI-specific tools were used; most often, results of the SF-36 tool were reported. Other tools were used in 1–3 records each (Fig. 3). Most records provided insights on the physical (*n = *28) and mental health (*n = *23) of adults with OI. Additionally, pain (*n = *7), fatigue (*n = *5) and social functioning (*n = *6) were described (Fig. 3). A detailed narrative synthesis of this topic is documented in Table 5.

**Table 5:** Narrative synthesis of included records on the HRQoL in adults with OI

domain	N	Findings	Records
Physical function	28	Physical functioning was described using a variety of tools however SF-36 (*n = *13) was used most often. Included records suggest that physical functioning and independence are lower in adults with OI compared with the overall population. In all included records (*n = *4), sleep was affected in individuals with OI	[31, 35, 52, 103, 109, 111, 151, 180, 201, 205, 206, 253–267]
Pain	7	Records concluded that adults with OI experienced significantly more pain and pain interference compared with the overall population	[13, 52, 106, 187, 254, 265, 268]
Fatigue	5	Included records concluded that individuals with OI experienced significantly more fatigue compared with the overall population, however women were overrepresented (62–78% of study samples)	[13, 106, 187, 254, 269]
Mental health	23	Included records on the mental health of adults with OI reached conflicting conclusions on the effect of OI on individuals’ mental health	[13, 35, 52, 106, 109, 111, 151, 205, 206, 253–262, 265–267, 270]
Social functioning	6	Records using validated tools (*n = *4) found that social functioning of adults with OI was comparable with the overall population	[13, 106, 109, 206, 266, 267]

*HRQoL* Health-related quality of life, *OI* Osteogenesis imperfecta, *SF-36* Short Form Questionnaire

### HRQoL of children with OI

Of 51 records (13 abstracts, 38 full texts) of 48 unique studies that described the HRQoL of children with OI, 41 records (80.4%) included children and 10 (19.6%) included a mixed population. In those records providing the sex of participants (*n = *44), the proportion of male and female participants was balanced (median: 50.0%).

**Fig. 4:**
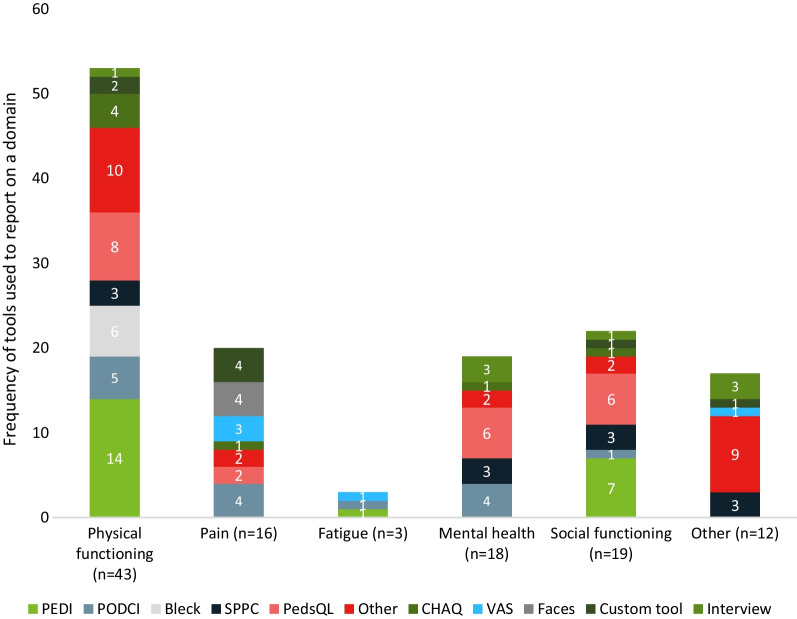
Tools used to assess paediatric HRQoL domains and number of records on each domain. *CHAQ* Childhood Health Assessment Questionnaire, *HRQoL* Health-related quality of life, *PEDI* Paediatric Evaluation of Disability Inventory, *PedsQL* Paediatric Quality of Life Inventory, *PODCI* Paediatric Outcomes Data Collection Instrument, *SPPC* Self-Perception Profile for Children, *VAS* Visual Analogue Scale

A variety of tools were used, none of which were OI specific (Fig. 4). Records focused predominantly on physical functioning (*n = *43); fewer records included data on social functioning (*n = *16), pain (*n = *16) and mental health (*n = *18) (Fig. 4). Few focused on fatigue (*n = *3) and other domains, including cognition, speech, physical appearance, dyspnoea, overall wellbeing, eating habits, care experience and barriers to physical activity (*n = *12). A detailed narrative synthesis of this topic is documented in Table 6.

**Table 6:** Narrative synthesis of included records on the HRQoL in children with OI

domain	N	Findings	Records
Physical function	42	Physical functioning tools mostly assessed mobility, athleticism, and function/independence. Physical functioning and mobility of children with OI were found to significantly differ from reference or control groups and between OI types	[37, 104, 106–108, 110, 152, 163, 164, 180, 188, 204, 213, 240, 271–298]
Pain	15	Few comparisons of pain experienced by children with OI compared with other children have been conducted. Children with OI experience fluctuating pain with bisphosphonate treatment cycles. Differences between fracture and non-fracture pain are poorly understood	[106, 107, 271, 276, 279, 280, 282–284, 286, 288, 290, 294, 295, 299]
Fatigue	3	Fatigue in children with OI is poorly understood, but included records indicate that children with OI do not experience significantly more fatigue compared with other children	[106, 276, 300]
Mental health	18	Few records compared the emotional functioning of children with OI to a reference or control population, therefore no consensus was identified	[48, 106, 110, 163, 204, 274, 276, 281–287, 289, 294, 301, 302]
Social functioning	19	Children with OI may experience impaired social functioning possibly due to the need for careful play and inability to participate in activities. Social functioning may be worse in children with more severe OI types compared with those with mild types	[48, 106, 108, 110, 163, 164, 204, 272–276, 284–287, 289, 294, 297]
Other	12	Other assessed domains in included records were cognition, speech, physical appearance, dyspnoea, overall wellbeing, eating habits, care experience and barriers to physical activity. Notable findings included the high prevalence of choosy eating in children with OI and high prevalence of food fussiness	[48, 107, 271, 274, 276, 284, 301–306]

*HRQoL* Health-related quality of life, *OI* Osteogenesis imperfecta

### HRQoL of caregivers of individuals with OI

**Fig. 5:**
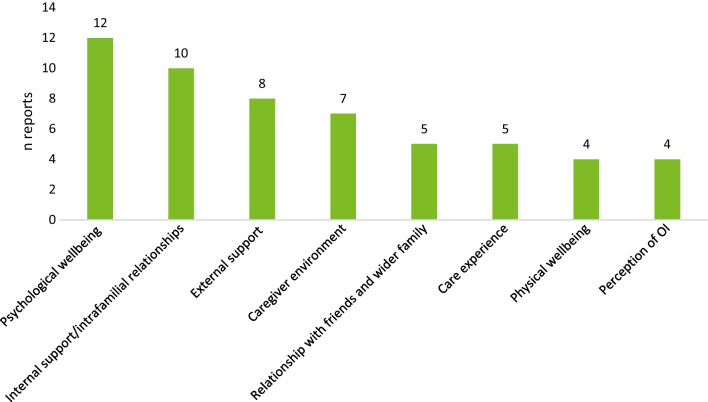
Domains examined by records on caregivers’ HRQoL. *HRQoL* Health-related quality of life, *OI* Osteogenesis imperfecta

Of 17 records of 16 unique studies (3 abstracts, 14 full texts) 14 included the sex of participants. Across such records, most participants were female (median: 66.7%). Most caregivers were either mothers or fathers to the care recipients. Two records included 4 siblings total [48, 49]. Care recipients in all studies were children; one study additionally reported on caregivers of 3 young adults (21–30 years of age) [50]. Records discussed themes of psychological wellbeing, familial and external support and relationships, care experience, physical wellbeing, and caregivers’ perception of OI (Fig. 5). The detailed narrative synthesis of this topic is documented in Table 7.

**Table 7:** Narrative synthesis of included records on the HRQoL of caregivers for individuals with OI

domain	N	Findings	Records
Psychological wellbeing	12	The OI diagnosis may affect the mental health of caregivers negatively. Parents of children with OI scored significantly lower in domains assessing mental health in studies that used validated tools	[48–50, 104, 290, 294, 307–312]
Internal support	10	Parents mentioned that having a child with OI may affect intrafamilial relationships and help from close family may be limited due to safety concerns of parents	[48–50, 54, 290, 294, 308–310, 313]
External support	8	Caregivers mentioned that more reliable and safe respite care opportunities for children with OI are important to facilitate caregivers’ wellbeing. Levels of institutional support received by families were higher if children were diagnosed with a more severe OI type	[48, 50, 308–311, 313, 314]
Caregiver environment	7	Caregivers of children with OI may experience a decrease in their household income, higher rates of absenteeism, significant medical care expenses for their child and may have to relocate to accommodate medical and safety needs	[48, 50, 54, 60, 307–310]
Relationships with friends	5	Parents noted that they may experience isolation from their social circle due to safety concerns for their child and a lack of understanding for the condition from outside parties	[307, 308, 310–312]
Care experience	5	Important elements of providing care to children with OI include coordinating medical care, assisting with ADLs and transfers, research of best care practices, advocacy, and adaptation of the home environment to be safer for children with OI	[48, 54, 310, 311, 313, 315]
Physical wellbeing	4	Findings are conflicting in the included records. Some parents reported experiencing recurring health issues after their child’s diagnosis	[307–310]
Perception of OI	4	In the included records, engagement with healthcare providers was affected by caregivers’ negative perception of their children's OI diagnosis	[49, 309, 310]

*ADLs* Activities of daily living, *HRQoL* Health-related quality of life, *OI* Osteogenesis imperfecta

### Economic outcomes of individuals with OI

Economic data were included in 11 records of 11 unique studies (2 abstracts, 9 full texts) [39, 51–60]. All featured data on the economic impact of OI, but only 7 were specific to the condition [39, 51, 53, 55, 56, 59, 60]. Seven records included children, and 2 each included adults or mixed populations. Most reported US or UK data and provided information on resource utilisation (*n = *7) [39, 51, 53, 55, 57–59], direct medical costs (*n = *10) [51–60], such as treatment and hospitalisation costs, and indirect medical costs (*n = *2) [57, 60], such as out of pocket expenses and travel expenses. Few records included direct costs beyond hospitalisation-associated expenditure and resource utilisation. None included information on costs associated with co-payments or home modifications.

### Quality assessment of the included studies

**Fig. 6:**
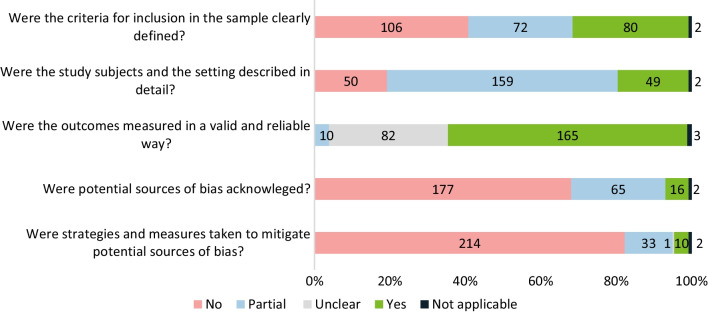
Quality assessment of records including clinical, humanistic, and economic data (*n = *260) following a modified JBI checklist for cross-sectional studies

The majority of the included records assessed according to the JBI fell short of fulfilling all requirements (Fig. 6): for 68.5%, inclusion criteria were not reported (*n = *106, 40.8%) or reported partially (*n = *72, 27.7%). Most records (*n = *159, 61.2%) provided partial descriptions of the study setting and subjects. For 31.5% (*n = *82) the validity of the employed outcome measures was unclear. Notably, in most records, potential bias sources were not acknowledged 68.1% (*n = *177) and in a further 25.0% (*n = *65) they were only acknowledged partially; in most instances no strategies were employed to mitigate bias (82.3%, *n = *214).

**Fig. 7:**
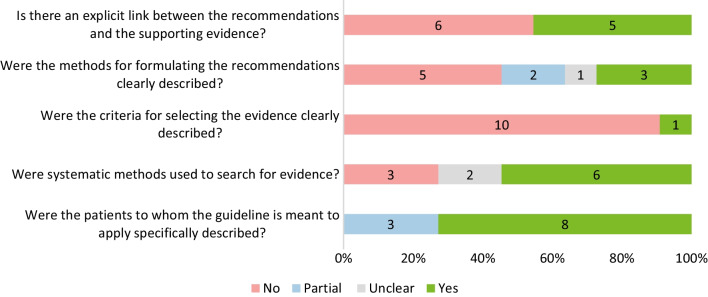
Quality assessment of treatment and management guidelines for OI (*n = *11) following the AGREE II checklist

Among the 11 included guides for clinical practice in OI (Fig. 7), 54.5% (*n = *6) did not provide explicit links between recommendations and supporting evidence, and 45.5% (*n = *5) did not describe methods with which recommendations were formulated clearly. Furthermore 90.9% (*n = *10) did not provide clear criteria for included evidence.

## Discussion

This systematic scoping review provides first comprehensive overview of the available literature on the impact of OI on individuals with OI, their families, caregivers, and wider society, including clinical, humanistic, and economic data and can therefore inform future research directions.

Existing reviews of OI literature do not provide a systematic overview of the scope of published records, but rather follow a narrative design [61–63], or aim to answer specific research questions [9, 11, 18–21, 64–72]. Therefore, the aim of this review was to determine the breadth of literature available and provide a snapshot of the evidence it covers. With this approach, data gaps could be identified to guide the direction of future studies.

This work finds that, while the high number of identified records suggests a high research interest into OI, many aspects of the condition that affect individuals, caregivers and healthcare systems are currently insufficiently documented or understood. The quality assessment of included records found that most records did not identify, or address, bias and a considerable number did not describe inclusion criteria for study participants and evidence or included samples. This limitation is persistent across research topics and constrains the generalisability of study findings.

Additionally, the high proportion of cross-sectional study designs, reporting inconsistencies across studies, and the predominance of records from Northern America and Northern Europe hinders our understanding of the global impact of OI for individuals, families and healthcare systems.

Choosing a scoping approach allowed us to capture the breadth of evidence on OI, both quantitative and qualitative, thereby allowing us to identify some rarely reported research. However, the diversity and high number of included records limited the depth of analysis we were able to undertake. Additionally, the focus on English language records presents a limitation which we have attempted to mitigate through the inclusion of a wide variety of databases.

While some clinical conditions, such as bone-related events and conditions are well-documented, others that may negatively affect the HRQoL of individuals with OI, are often not covered in the literature. This systematic scoping review uncovered limited information on women’s health, treatment-related adverse events, and pulmonary-, GI-, kidney-, sleep-, and skin-related conditions.

Recent publications underline the importance of research into the conditions identified as data gaps in this field: in one study individuals reported that their HRQoL has been affected by urinary tract, skin, GI, and neurological conditions [13]. Additionally, other studies point to pulmonary conditions being among the most commonly reported causes of death in individuals with OI [73, 74]. Furthermore, understanding the benefits and adverse effects of treatments for individuals with rare metabolic conditions has been identified as a priority research question in a joint collaboration of patients, carers and healthcare professionals [75].

We identified few records of interactions with the healthcare system, the majority of which included data from North-western Europe or Northern America. Of those, few records included patients’ experience with services and their progression through the healthcare system. In records that described interactions with healthcare professionals, few described genetic testing, outpatient care, operative interventions, and dental care. Similarly, few included records included information on prenatal testing, blood and DNA analysis, and misdiagnosis or diagnostic uncertainty. The monitoring of individuals with OI or ongoing care was not well documented. Similarly, guidance on most care topics for individuals with OI is limited and often unspecific to OI.

HRQoL is well documented for individuals with OI, however more records of women and children, especially with milder OI types, were identified, while fewer records of young adults, men, and those with OI types 3 and 4 were found. A variety of tools were applied in the studies, which limits our ability to compare and generalise findings across studies. Few adult studies used tools that were specific to long-term disability, pain, or fatigue associated with long-term conditions, but physical and mental functioning were well described. Among paediatric records, use of disability-specific tools to assess physical functioning was prevalent, however, pain, fatigue, and mental health in children with OI were not well described.

Records on the HRQoL of caregivers featured a high number of interview-based studies and limited documentation of caregiver- and care-recipient characteristics, which may hinder the generalisability of study findings. Furthermore, few records included fathers, siblings and other family members or families of young adult and adult care recipients.

Few records included data on the economic impact of OI on individuals, healthcare systems and wider society. Most focussed on hospitalisation and associated costs, whereas indirect costs, and outpatient care consumption and costs are less well documented. Therefore, the identified data does not allow an accurate assessment of OI-associated costs and expenses.

Patient groups are unequally represented across outcomes assessed in this review: more records described clinical conditions, current care practice and HRQoL in children; similarly, OI specific clinical practice guidelines were mostly available for children or adolescents. In patient HQoL studies adult men, adolescents and older adults were underrepresented. Few studies provided data on the wellbeing of male caregivers or family members of individuals with OI. The lack of evidence for these population groups compromises any attempts at evidence-based care and is well documented to decrease the generalisability of findings, quality of care and hinder the access to effective interventions [76].

## Conclusion

This work shows that despite the interest of the research community and the persistent patient need, many research areas remain to be explored to better understand the impact of OI and accurately depict individuals’ experiences. Among such gaps, future research into health concerns beyond bone health, the long-term effects of OI treatment and changing medical needs throughout an individual’s life can help to better understand and care for individuals with the condition. Additionally, a better understanding of pain and fatigue experiences, as well as the HRQoL of caregivers and families affected by OI, can aid in planning and directing services. Lastly, OI may pose a considerable economic burden to individuals and society; however, few records assess the costs associated with OI treatment and care outside of the hospital setting. An in-depth documentation of such costs could help to address concerns of affected individuals and their families.

Funding and resources for research on rare diseases are limited. However, this review represents a first step to mitigate data paucity through the identification of specific research gaps within OI. Based on these gaps we have designed and conducted an online-based survey targeted at individuals with OI, their caregivers, and close relatives [77]. We hope that the findings from the survey will help the healthcare community gain insights into the clinical, economic, and humanistic burden of this condition.

## Supplementary Information

**Additional file 1.** Supplementary tables.

## Data Availability

Not applicable.

## Abbreviations

ADL: Activities of daily living
AGREE: Appraisal of guidelines research evaluation
APEG: Australasian paediatric endocrine group
BMI: Body mass index
CEA: Center for the evaluation of value and risk in health
CENTRAL: Cochrane central register of controlled trials
CHAQ: Childhood health assessment questionnaire
CRD: Centre for reviews and dissemination
DEXA: Dual-energy X-ray absorptiometry
EMQN: European molecular genetics quality network
EQ-5D-5L: Euroqol 5-dimension questionnaire 5 levels
FIM: Functional independence measure
GI: Gastrointestinal
HCP: Health care professional
HCRU: Healthcare resource use
HRQoL: Health-related quality of life
JBI: Joanna Briggs Institute
MoD: Mode of delivery
NHS EED: National health service economic evaluation database
OI: Osteogenesis imperfecta
PEDE: Paediatric economic database evaluation
PEDI: Paediatric evaluation of disability inventory
PedsQL: Paediatric quality of life inventory
PODCI: Paediatric outcomes data collection instrument
PRISMA: Preferred reporting items for systematic reviews and meta-analyses
PROMIS: Patient-reported outcomes measurement
PROSPERO: International prospective register of systematic reviews
RCT: Randomised controlled trial
ScHARRHUd: School of health and related research utilities database
SF-36: Short Form 36
SPPC: Self-perception profile for children
VAS: Visual analogue scale
