# Supplementary material for: The patient clinical journey and socioeconomic impact of osteogenesis imperfecta: a systematic scoping review
Source: Orphanet J Rare Dis. 2023 Feb 22;18:34. doi: 10.1186/s13023-023-02627-3 (PMC9945474; doi:10.1186/s13023-023-02627-3)
Supplement: Supplementary file 1 — Additional file 1. Supplementary tables. [file 13023_2023_2627_MOESM1_ESM.docx]

# Supplementary materials

## Search terms

Supplementary Table 1 Search terms of database searches conducted 11 December 2020–18 December 2020

| Set number | | | Search term |  |
| --- | --- | --- | --- | --- |
| Embase search: 11.12.2020 | | | | |
| #1 | | | 'osteogenesis imperfecta'/de | |
| #2 | | | 'osteogenesis imperfecta':ti,ab,kw | |
| #3 | | | 'brittle bone disease':ti,ab,kw | |
| #4 | | | glassbone:ti,ab,kw | |
| #5 | | | 'glass bone':ti,ab,kw | |
| #6 | | | #1 OR #2 OR #3 OR #4 OR #5 | |
| #7 | | | (#1 OR #2 OR #3 OR #4 OR #5) AND [english]/lim | |
| #8 | | | 'editorial'/de OR 'letter'/de | |
| #9 | | | 'case report'/de | |
| #10 | | | #8 OR #9 | |
| #11 | | | #7 NOT #10 | |
| MEDLINE/Pubmed: 12.12.2020 | | | | |
| #1 | Search: (ENGLISH[Language] AND (("osteogenesis imperfecta"[Title/Abstract] OR "osteogenesis imperfecta"[MeSH Terms] OR "osteogenesis imperfecta"[Other Term] OR "brittle bone disease"[Title/Abstract] OR "brittle bone disease"[Other Term]) OR (glassbone [tiab] OR glassbone [OT] OR "glass bone" [tiab] OR "glass bone" [ot]))) NOT ((EDITORIAL[Publication Type]) OR (LETTER[Publication Type]) OR (COMMENT[Publication Type]) OR ('CASE REPORT' [TI]) OR (ANIMALS [MH] NOT HUMANS [MH])) Saved search **Sort by**: Most Recent | | | |
| Cochrane Central Register of Controlled Trials (CENTRAL): 12.12.2020 | | | | |
| #1 | "osteogenesis imperfecta":ti,ab,kw | | | |
| #2 | MeSH descriptor: [Osteogenesis Imperfecta] this term only | | | |
| #3 | "brittle bone disease":ti,ab,kw | | | |
| #4 | glassbone:ti,ab,kw | | | |
| #5 | "glass bone":ti,ab,kw | | | |
| #6 | #1 OR #2 OR #3 OR #4 OR #5 in Trials | | | |
| PsycINFO: 15.12.2020 | | | | |
| #1 | "osteogenesis imperfecta" OR “brittle bone disease” OR glassbone OR “glass bone” | | | |
| #2 | #1 OR su.exact("osteogenesis imperfecta") | | | |
| #3 | #2 AND LA(english) | | | |
| #4 | SU.EXACT("Literature Review") OR SU.EXACT("Systematic Review") OR SU.EXACT("Case Report") | | | |
| #5 | #3 NOT #4 | | | |
| National Health Service Economic Evaluation Database (NHS EED): 12.12.2020 | | | | |
| #1 | MeSH DESCRIPTOR Osteogenesis Imperfecta EXPLODE ALL TREES | | | |
| #2 | (osteogenesis imperfecta) in NHSEED | | | |
| #3 | (osteogenesis imperfecta) OR (brittle bone) OR (glassbone OR glass bone) IN NHSEED | | | |
| #4 | #1 OR #2 OR #3 | | | |
| Centre for the Evaluation of Value and Risk in Health (CEA registry): 12.12.2020 | | | | |
| #1 | | osteogenesis | | |
| #2 | | imperfecta | | |
| #3 | | brittle | | |
| #4 | | glass | | |
| Paediatric Economic Database Evaluation (PEDE): 12.12.2020 | | | | |
| #1 | Keywords: (TITLE_ABSTRACT_KEYWORDS "osteogenesis(-\| )imperfecta")  Age groups: Perinates Neonates Infants Children Adolescents  Years: 1980 - 2019 | | | |
| #2 | Keywords: (TITLE_ABSTRACT_KEYWORDS "brittle(-\| )bone")  Age groups: Perinates Neonates Infants Children Adolescents  Years: 1980 - 2019 | | | |
| #3 | Keywords: (TITLE_ABSTRACT_KEYWORDS "glass(-\| )bone")  Age groups: Perinates Neonates Infants Children Adolescents  Years: 1980 - 2019 | | | |
| School of Health and Related Research Utilities Database (ScHARRHUd): 12.12.2020 | | | | |
| #1 | imperfecta OR brittle OR glass bone | | | |
| Orphanet ^a^: 12.12.2020 | | | | |
| #1 | osteogenesis imperfecta | | | |
| #2^a^ | Lobstein disease [ti] OR Lobstein’s disease [ti] | | | |
| Google Scholar ^b^: 18.12.2020 | | | | |
| #1 | “osteogenesis imperfecta” and year 2020 | | | |
| #2 | “osteogenesis imperfecta” and year 2019 | | | |
| #3 | “osteogenesis imperfecta” and year 2018 | | | |
| #4 | “osteogenesis imperfecta” and year 2017 | | | |
| #5 | “osteogenesis imperfecta” and year 2016 | | | |
| #6 | “osteogenesis imperfecta” and year 2015 | | | |
| #7 | “osteogenesis imperfecta” and year 2014 | | | |
| #8 | “osteogenesis imperfecta” and year 2013 | | | |
| #9 | “osteogenesis imperfecta” and year 2012 | | | |
| #10 | “osteogenesis imperfecta” and year 2011 | | | |

^a^ The Orphanet search included terms not used in MEDLINE (Pubmed) searches, but also omitted terms that were used in previous searches.^b^ Google Scholar was searched using the Publish or Perish software (https://harzing.com/resources/publish-or-perish). Publish or Perish launches searches onto Google Scholar and offers the option to download the results of the searches in EndNote format. Due to the Google Scholar limit of 1000 records per download searches were conducted by year.

ab, abstract; de, no explode, retrieves only the term specified and no headings below it; kw, keyword; lim, limit, limits search; ti, title.

## Eligibility criteria

Supplementary Table 2 Problem/population, intervention, comparator/control, outcome, study design (PICOS) elements

| P | Patient or problem | 1. Clinical impact/patient journey    - Adults ^a^ and children ^b^ with OI 2. Humanistic impact    - Adults ^a^ and children ^b^ with OI    - Families and caregivers of people with OI 3. Economic impact    - Adults ^a^ and children ^b^ with OI    - Families and caregivers of people with OI    - Wider society |
| --- | --- | --- |
| I | Intervention | No specified intervention |
| C | Control or comparison | No specified comparator |
| O | Outcome | 1. Clinical impact/patient journey   Primary   - - Key clinical events and health conditions   - Wider health concerns beyond fractures   - Equity concerns   - Socio-economic mediators for access to treatment   - Diagnosis and monitoring   - Interactions within the healthcare system   Secondary ^c^   - - Information on standard of care   - Off-label prescribing   - Country-specific treatment approaches   - Characteristics of sub-populations  1. Humanistic impact    - Disease-specific HRQoL outcomes    - Generic HRQoL outcomes    - Utility measures    - Factors affecting HRQoL    - Patient reported outcomes 2. Economic impact    - Direct healthcare costs    - Indirect healthcare costs    - Healthcare resource use    - Non-healthcare costs |
| S | Study design | 1. Clinical impact/patient journey    - Clinical guidelines    - Patient registry data    - Patient and healthcare provider surveys    - Cohort studies (≥50 patients) ^d^    - Cross-sectional studies (≥50 patients) ^d^    - Case-control studies (≥50 patients) ^d, e^ 2. Humanistic and economic impact    - RCTs    - Non-RCTs    - Cohort studies    - Patient registry data    - Patient surveys    - Cross-sectional studies    - Case-control studies    - Case series (≥10 patients) ^d^    - Economic evaluations ^f^    - HCRU/cost studies ^g^ |
|  | Record type | - - Manuscripts   - Conference proceedings   - Grey literature ^h^ |
|  | Date | 1 January 1995 to 18 December 2020^i^ |
|  | Language | English |
|  | Country | No restriction |
| ^a^ Defined as individuals ≤18 years unless stated otherwise by authors; ^b^ Defined as individuals ≥19 years unless stated otherwise by authors; ^c^ Secondary outcomes were captured and reported as a top line summary narrative; ^d^ Larger studies assumed more likely to have a protocol and provide higher quality evidence; ^e^ Case-control studies were excluded if the case group included <50 patients; ^f^ Cost-minimisation analyses, cost-effectiveness analyses, cost-utility analyses, cost-benefit analyses; ^g^ Registry and chart reviews, real-world data; ^h^ Supplementary, fully documented, internet searches of grey literature were conducted using Google Scholar and Publish or Perish software; ^I^ searches were conducted from 11 -18 December, for specific dates see Table 8  HCRU, healthcare resource utilisation; HRQoL, health-related quality of life; OI, osteogenesis imperfecta; RCT, randomised controlled trial | | |

## Quality assessment tools

Supplementary Table 3 Custom assessment tool designed based on the JBI checklist for cross-sectional studies

| Assessment question | Description |
| --- | --- |
| Were the criteria for inclusion in the sample clearly defined? | Criteria include OI type, age, gender, disease characteristics, medications, socioeconomic status, education, living situation as is relevant to the particular study aims. |
| Were the study subjects and the setting described in detail? | Can include descriptions of people with OI, children, caregivers. Factors include OI type, age, gender, disease characteristics, medications, socioeconomic status, education, living situation as is relevant to the particular study aims. |
| Were the outcomes measured in a valid and reliable way? | This category considers whether outcomes were assessed using existing definitions, diagnostic criteria, or validated tools. Additionally, this category should consider whether outcomes are observer or self-reported. It should also be considered whether the measurement tools used are validated instruments for the purpose used in the record and how the measurements were conducted (e.g., as part of an RCT, by 1 or more scientists, in a structured manner). |
| Were potential sources of bias acknowledged? | Considers whether the authors describe and identify limitations in the record and critically appraise the methodology and findings. It should be considered whether the authors describe influences or factors outside of the study, that may influence or bias the direction or interpretation of the results.  Typically, such factors include baseline characteristics, prognostic factors, treatments, living situation.  To answer this question, it should also be considered whether the study sample is representative of OI types, sex, family members and countries. |
| Were strategies and measures taken to mitigate potential sources of bias? | Here it should be considered whether the authors addressed the sources of bias they identified (if indeed any sources were acknowledged).  Strategies to address the effects of biasing factors may be included within the study design or in the data analysis. Bias may be addressed, for example, by matching or stratifying sampling of participants, applying exclusion criteria, and performing regression analyses and/or other statistical approaches to analysis. |

Abbreviation: JBI, Joanna Briggs institute; OI, osteogenesis imperfecta; RCT, randomised controlled trial.

## Included records

Supplementary Table 4 Included records containing clinical data

| Reference | Conditions | Current practice | Guidance | Diagnosis | Monitoring | Healthcare interactions |
| --- | --- | --- | --- | --- | --- | --- |
| Aarabi, Rauch (142) | X |  |  | X |  |  |
| Aglan, Zaki (164) | X |  |  |  |  |  |
| Ahn, Carter (139) | X |  |  |  |  |  |
| Al Agha, Qari (124) | X |  |  |  |  |  |
| Alaei, Mosallanejad (220) | X |  |  |  |  |  |
| Andersson, Dahllöf (194) | X |  |  |  |  |  |
| Anissipour, Hammerberg (136) | X | X |  |  |  |  |
| Antoniazzi, Mottes (47) |  |  | X |  |  |  |
| Apolinário, Sindeaux (193) | X | X |  |  |  |  |
| Arponen (156) | X | X |  | X |  |  |
| Arponen, Mäkitie (152) | X | X |  | X |  |  |
| Bains, Carter (128) | X | X |  |  |  |  |
| Barber, Abbott (170) | X |  |  |  |  |  |
| Bellur, Jain (40) | X | X |  | X |  | X |
| Ben Amor, Roughley (130) | X | X |  |  |  |  |
| Bianchi, Leonard (239) |  |  | X |  |  |  |
| Binh, Maasalu (88) | X | X |  | X |  |  |
| Brizola, Staub (208) | X | X |  |  |  |  |
| Brizola, Zambrano (151) | X |  |  | X |  |  |
| Byers, Krakow (240) |  |  | X |  |  |  |
| Castro, Ribeiro (165) | X |  |  |  |  |  |
| Castro, Santos (87) | X | X |  | X |  |  |
| Charnas and Marini (96) | X |  |  |  |  |  |
| Chetty, Roberts (205) | X |  |  |  |  |  |
| Cheung, Arponen (131) | X | X |  |  |  |  |
| Cianferotti and Brandi (241) |  |  | X |  |  |  |
| Clark, Burren (36) |  |  |  |  |  | X |
| Cubert, Cheng (234) |  | X |  | X | X | X |
| da Costa Otavio, Teixeira (215) | X |  |  |  |  |  |
| Daly, Wisbeach (209) | X | X |  |  |  |  |
| Dar, Khalily (29) | X | X |  | X |  |  |
| de Graaff, Verra (38) |  | X |  |  |  | X |
| de Lima, de Lima (133) | X |  |  |  |  |  |
| DeVile, Allgrove (154) | X | X |  |  |  |  |
| Engelbert, Uiterwaal (95) | X | X |  |  |  |  |
| Engelbert, Uiterwaal (314) | X |  |  |  |  |  |
| Fassier, Rauch (144) | X |  |  |  |  |  |
| Feinstein, Shapiro (212) | X |  |  |  |  |  |
| Folkestad, Hald (74) | X |  |  |  |  |  |
| Folkestad, Hald (221) | X |  |  |  |  |  |
| Folkestad, Hald (118) | X |  |  |  |  |  |
| Galindo-Zavala, Bou-Torrent (242) |  |  | X |  |  |  |
| Galloway, Nixon (41) |  |  |  |  |  | X |
| Germain-Lee, Brennen (166) | X | X |  |  |  |  |
| Gjørup, Bendixen (195) | X |  |  |  |  |  |
| Gjørup, Hald (196) | X |  |  |  |  |  |
| Gjørup, Hald (177) | X |  |  |  |  |  |
| Glorieux and Rauch (228) | X |  |  |  |  |  |
| Goeller, Esposito (232) | X | X |  |  |  |  |
| Goudriaan, Harsevoort (76) | X | X |  |  |  |  |
| Graff and Syczewska (171) | X |  |  |  |  |  |
| Graff, Kalinowska (138) | X |  |  |  |  |  |
| Greeley, Donaruma-Kwoh (115) | X |  |  | X |  |  |
| Hagberg, Lowing (33) |  |  |  | X | X | X |
| Hald, Folkestad (78) | X |  |  |  |  |  |
| Hald, Folkestad (112) | X | X |  |  |  |  |
| Hald, Folkestad (94) | X | X |  |  |  |  |
| Hatz, Esposito (90) | X |  |  |  |  |  |
| Hernández Jiménez, Saavedra Falero (181) | X |  |  |  |  |  |
| Hill, Murphy (233) | X |  |  |  |  |  |
| Hoseinbeyki, Moradifard (79) | X |  |  |  |  |  |
| Hupin, Edwards (80) | X |  |  |  |  |  |
| Jain, Tam (172) | X | X |  | X |  |  |
| Janus, Engelbert (146) | X |  |  |  |  |  |
| Jensen and Lund (167) | X |  |  |  |  |  |
| Kadhim, Holmes (81) | X | X |  |  |  |  |
| Kallur, Kruse (125) | X |  |  |  |  |  |
| Kok, Sakkers (163) | X |  |  | X |  |  |
| Koumakis, Dellal (117) | X |  |  |  |  |  |
| Kovero, Pynnönen (147) | X |  |  |  |  |  |
| Kuurila, Kaitila (188) | X |  |  |  |  |  |
| Li, Lyu (113) | X |  |  |  |  |  |
| Li, Rush (219) | X |  |  |  |  |  |
| Li, Xia (97) | X | X |  |  |  |  |
| Lin, Chuang (120) | X |  |  |  |  |  |
| Lindahl, Kindmark (111) | X | X |  |  |  |  |
| Lindahl, Rubin (126) | X |  |  |  |  |  |
| Liu, Asan (235) | X |  |  | X |  |  |
| Lund, M (189) | X |  |  |  |  |  |
| Ma, S (190) | X |  |  |  |  |  |
| Machol, Hadley (214) | X |  |  |  |  |  |
| Maioli, Gnoli (137) | X |  |  |  |  |  |
| Malmgren and Norgren (200) | X |  |  |  |  |  |
| Malmgren, Andersson (207) | X |  |  |  |  |  |
| Martin, Haney (100) | X |  |  | X |  |  |
| Mata Caballero, Hernandez Jimenez (173) | X |  |  |  |  |  |
| Mata Caballero, Hernandez Jimenez (222) | X |  |  |  |  |  |
| McAllion and Paterson (73) | X |  |  |  |  |  |
| McAllion and Paterson (160) | X | X |  |  |  |  |
| Michalus, Nowicka (231) | X | X |  |  |  |  |
| Moreira, Gilbert (42) |  |  |  | X |  | X |
| Moreira¹, Angelica (43) | X |  |  | X |  | X |
| Mueller, Engelbert (243) |  |  | X |  |  |  |
| Munns, Rauch (110) | X | X |  |  |  |  |
| Narayanan, Dougan (34) | X | X |  | X | X | X |
| Nguyen, Saag (197) | X |  |  |  |  |  |
| Oduah (82) | X | X |  |  |  |  |
| Oduah, Firth (83) | X | X |  | X |  |  |
| Ohata, Takeyari (119) | X |  |  | X |  |  |
| Okawa, Kubota (198) | X | X |  |  |  |  |
| Oliveira, Peters (98) | X | X |  |  |  |  |
| Palomo, Glorieux (174) | X | X |  |  |  |  |
| Pasieka, Kuhn (225) | X |  |  |  |  |  |
| Patel, Nagamani (121) | X | X |  | X |  |  |
| Paterson, Monk (315) | X |  |  |  |  |  |
| Paterson, Ogston (229) | X |  |  |  |  |  |
| Payet and Cormier (89) | X | X |  |  |  |  |
| Peddada, Sullivan (92) | X | X |  |  |  |  |
| Pedersen (216) | X | X |  |  |  |  |
| Petersen and Wetzel (206) | X |  |  |  |  |  |
| Philip and Qadri (224) | X |  |  |  |  |  |
| Pinheiro, Barrios (182) | X |  |  | X |  |  |
| Radunovic and Steine (218) | X |  |  |  |  |  |
| Radunovic, Wekre (168) | X |  |  |  |  |  |
| Radunovic, Wekre (140) | X |  |  |  |  |  |
| Rauch, Lalic (132) | X |  |  | X |  |  |
| Rauch, Plotkin (129) | X | X |  |  |  |  |
| Rauch, Robinson (179) | X | X |  |  |  |  |
| Rodrigues, Chianca (175) | X |  |  |  |  |  |
| Rodriguez Celin, Kruger (103) | X | X |  |  |  |  |
| Rossini, Adami (244) |  |  | X |  |  |  |
| Ruggiero, Dodson (245) |  |  | X |  |  |  |
| Rush, Li (180) | X |  |  |  |  |  |
| Rusinska and Michalus (84) | X |  |  |  |  |  |
| Rusinska, Jakubowskapietkiewicz (153) | X |  |  | X |  |  |
| Salter, Offiah (227) |  | X |  |  |  |  |
| Sato, Ouellet (159) | X | X |  |  |  |  |
| Scheres (85) | X |  |  |  |  |  |
| Scheres, van Dijk (122) | X | X |  |  |  |  |
| Semler, Hoyer-Kuhn (176) | X |  |  |  |  |  |
| Semler, Hoyer-Kuhn (184) | X | X |  |  |  |  |
| Semler, Cheung (158) | X | X |  |  |  |  |
| Sepúlveda, Terrazas (32) | X | X |  | X | X |  |
| Shapiro and Germain-Lee (246) |  |  | X |  |  |  |
| Simm, Biggin (247) |  |  | X |  |  |  |
| Simoes, Fernandes (226) | X |  |  | X |  |  |
| Singer, Ogston (230) | X |  |  |  |  |  |
| Staun Larsen, Thuesen (191) | X |  |  |  |  |  |
| Stewart, Raja (30) | X |  |  | X |  | X |
| Swinnen, Coucke (217) | X |  |  |  |  |  |
| Swinnen, De Leenheer (91) | X | X |  |  |  |  |
| Swinnen, Dhooge (213) | X |  |  |  |  |  |
| Tabanfar (99) | X |  |  | X |  |  |
| Tam, Chen (145) | X | X |  |  |  |  |
| Tayne and Smith (93) | X | X |  |  |  |  |
| Thuesen, Gjørup (192) | X |  |  |  |  |  |
| To (134) | X |  |  |  |  |  |
| Tosi, Floor (210) | X | X |  | X |  |  |
| Trejo, Fassier (116) | X | X |  |  |  |  |
| van Brussel, van der Net (248) |  |  | X |  |  |  |
| van Dijk, Byers (249) |  |  | X |  |  |  |
| Veilleux, Lemay (169) | X |  |  |  |  |  |
| Violas, Fassier (143) | X |  |  |  |  |  |
| Vyskocil and Pavelka (236) |  |  |  | X |  |  |
| Wadanamby, Connolly (157) | X |  |  |  |  |  |
| Waltimo-Sirén, Kolkka (187) | X |  |  |  |  |  |
| Wekre, Eriksen (123) | X | X |  |  |  |  |
| Wekre, Kjensli (141) | X |  |  |  |  |  |
| White, White (250) |  |  | X |  |  |  |
| Yakhyaeva and Namazova-Baranova (86) | X | X |  |  |  |  |
| Yimgang and Shapiro (45) | X | X |  |  |  | X |
| Yimgang, Brizola (46) | X | X |  | X |  | X |
| Youngblom, Murray (237) |  |  |  | X |  |  |
| Zambrano, Brizola (183) | X | X |  |  |  |  |
| Zambrano, Brizola (127) | X | X |  |  |  |  |
| Zhytnik, Maasalu (135) | X |  |  |  |  |  |
| Zhytnik, Maasalu (114) | X |  |  |  |  |  |

Supplementary Table 5 Included records containing humanistic data

| Reference | Adult HRQoL | Paediatric HRQoL | Caregiver/ family HRQoL |
| --- | --- | --- | --- |
| Ablon (268) | X |  |  |
| Arabaci, Bozkurt (308) |  |  | X |
| Arponen, Bachour (263) | X |  |  |
| Ashby, Montpetit (276) |  | X |  |
| Ball, Roberts (294) |  | X |  |
| Balkefors, Mattsson (257) | X |  |  |
| Bernehäll Claesson and Brodin (312) |  |  | X |
| Bozkurt, Baysan Arabacı (307) |  |  | X |
| Bronheim, Khan (256) | X |  |  |
| Castro, Chougui (309) |  |  | X |
| Castro, Marinello (311) |  |  | X |
| Caudill, Flanagan (280) |  | X |  |
| Chevrel, Schott (266) | X |  |  |
| Dogba, Bedos (10) |  | X |  |
| Dogba, Rauch (310) |  |  | X |
| Dogba, Dahan-Oliel (50) |  |  | X |
| Elona, Mentari (283) |  | X |  |
| Engelbert, Custers (270) |  | X |  |
| Engelbert, Gulmans (272) |  | X |  |
| Engelbert, Uiterwaal (271) |  | X |  |
| Garganta, Jaser (286) |  | X |  |
| Gooijer, Harsevoort (251) | X |  |  |
| Graf, Hassani (279) |  | X |  |
| Haga, Kosaki (296) |  | X |  |
| Hagberg, Lowing (290) |  | X |  |
| Hald, Folkestad (253) | X |  |  |
| Harsevoort, Gooijer (267) | X |  |  |
| Hill, Baird (299) |  | X |  |
| Högler, Scott (293) |  | X |  |
| Hoyer-Kuhn, Stark (301) |  | X |  |
| Huang, Ambrose (281) |  | X |  |
| Jones, Theophile (303) |  | X |  |
| Keemink, Sakkers (304) |  | X |  |
| Khan, Yonko (258) | X |  |  |
| Kok, Sakkers (282) |  | X |  |
| Konstantynowicz and Abramowicz (295) |  | X |  |
| Lazow, Jaser (288) |  |  | X |
| Löwing, Aström (277) |  | X |  |
| Luiz and Coelho (273) |  | X |  |
| Marr (313) |  |  | X |
| Matsushita, Mishima (254) | X |  |  |
| Michalovic, Anderson (300) |  | X |  |
| Mills, Clark (302) |  | X |  |
| Montpetit, Dahan-Oliel (262) | X |  |  |
| Moshkovich, Benjamin (264) | X |  |  |
| Murali, Cuthbertson (278) |  | X |  |
| Nguyen, Thuy (289) |  | X |  |
| Nicolaou, Bowe (260) | X |  |  |
| Orlando, Pinedo-Villanueva (252) | X |  |  |
| Paiva (265) | X |  |  |
| Raimann, Pairitsch (285) |  | X |  |
| Rauch, Munns (275) |  | X |  |
| Rochmah and Faizi (287) |  | X |  |
| Rodríguez Celin and Fano (261) | X |  |  |
| Santos, Pires (49) |  |  | X |
| Seikaly, Kopanati (269) |  | X |  |
| Suskauer, Cintas (291) |  | X |  |
| Szczepaniak-Kubat, Kurnatowska (306) |  |  | X |
| Tolboom, Cats (274) |  | X |  |
| Tsimicalis, Boitor (284) |  | X |  |
| Van Brussel, Takken (298) |  | X |  |
| Vanz, Félix (305) |  |  | X |
| Widmann, Bitan (259) | X |  |  |
| Widmann, Laplaza (255) | X |  |  |
| Wiggins, Kreikemeier (292) |  | X | X |
| Wiggins and Kreikemeier (48) |  | X | X |
| Zack, Franck (297) |  | X |  |

Supplementary Table 6 Included records containing economic data

| Reference | Resource utilisation | Direct costs | Indirect costs |
| --- | --- | --- | --- |
| Darbà and Marsà (51) | X | X |  |
| Kolovos and Javaid (55) | X | X |  |
| Kreikemeier, Gosnell (58) | X | X |  |
| Meena, Panigrahi (56) |  | X |  |
| Saraff, Sahota (57) | X | X | X |
| Vitale, Matsumoto (59) | X | X |  |

Supplementary Table 7 Included records containing mixed outcomes

| Reference | Conditions | Current practice | Guidance | Diagnosis | Monitoring | Healthcare interactions | Adult HRQoL | Paediatric HRQoL | Caregiver/ family HRQoL | Resource utilisation | Direct costs | Indirect costs |
| --- | --- | --- | --- | --- | --- | --- | --- | --- | --- | --- | --- | --- |
| Amako, Fassier (150) | X |  |  |  |  |  |  | X |  |  |  |  |
| Arponen, Waltimo-Sirén (185) | X |  |  |  |  |  | X |  |  |  |  |  |
| Atta, Iqbal (105) | X | X |  |  |  |  |  | X |  |  |  |  |
| Aubry-Rozier, Richard (35) |  | X |  | X | X | X | X |  |  |  |  |  |
| Belyea and Knox (53) |  | X |  |  |  |  |  |  |  | X | X |  |
| Bendixen, Gjørup (203) | X |  |  |  |  |  | X |  |  |  |  |  |
| Coêlho, Luiz (211) | X |  |  |  |  |  |  | X |  |  |  |  |
| Dung, Armstrong (102) | X |  |  |  |  |  |  | X | X |  |  |  |
| Engelbert, Beemer (106) | X | X |  |  |  |  |  | X |  |  |  |  |
| Engelbert, van der Graaf (162) | X |  |  |  |  |  |  | X |  |  |  |  |
| Feehan, Zacharin (107) | X | X |  |  |  |  | X |  |  |  |  |  |
| Forestier-Zhang, Watts (52) |  |  |  |  |  |  | X |  |  |  | X |  |
| Gjørup, Beck-Nielsen (204) | X |  |  |  |  |  | X |  |  |  |  |  |
| Hald, Folkestad (109) | X |  |  |  |  |  | X |  |  |  |  |  |
| Hoyer-Kuhn, Semler (37) | X | X |  |  |  | X |  | X |  |  |  |  |
| Kruger, Caudill (178) | X | X |  |  |  |  | X | X |  |  |  |  |
| McKiernan (101) | X | X |  |  |  |  | X |  |  |  |  |  |
| Montpetit, Lafrance (186) | X | X |  |  |  |  |  | X |  |  |  |  |
| Murphy, Howard (60) |  |  |  |  |  |  |  |  | X |  | X | X |
| Najirad, Madathil (202) | X | X |  |  |  |  |  | X |  |  |  |  |
| Ruck, Dahan-Oliel (238) |  | X |  |  |  |  |  | X |  |  |  |  |
| Ruiter-Ligeti, Czuzoj-Shulman (39) | X |  |  |  |  | X |  |  |  | X |  |  |
| Rush, DeHaai (54) |  |  |  |  |  |  |  |  | X |  | X |  |
| Saeves, Lande Wekre (199) | X | X |  |  |  |  | X |  |  |  |  |  |
| Song, Zhao (161) | X | X |  | X |  |  |  | X |  |  |  |  |
| Tosi, Floor (104) | X | X |  | X |  |  | X | X |  |  |  |  |
| Tosi, Oetgen (13) | X | X |  |  | X | X | X |  |  |  |  |  |
| Vanz, van de Sande Lee (108) | X | X |  |  |  |  |  | X |  |  |  |  |
| Wekre, Frøslie (31) | X |  |  | X |  |  | X |  |  |  |  |  |
| Yonko, Emanuel (149) | X |  |  |  |  |  | X |  |  |  |  |  |
